# Regeneration and expansion of autologous full‐thickness skin through a self‐propagating autologous skin graft technology

**DOI:** 10.1002/ccr3.2533

**Published:** 2019-11-06

**Authors:** Charles W. Patterson, Matthew Stark, Silpa Sharma, Gerhard S. Mundinger

**Affiliations:** ^1^ Division of Plastic and Reconstructive Surgery Department of Surgery Louisiana State University Health Sciences Center New Orleans Louisiana; ^2^ Division of Plastic and Reconstructive Surgery Children’s Hospital of New Orleans New Orleans Louisiana; ^3^ Department of Pathology Children’s Hospital of New Orleans New Orleans Louisiana

**Keywords:** autologous tissue, dermis, epidermis, full‐thickness, hair follicle, hypodermis, melanin, skin regeneration

## Abstract

New autologous skin regeneration technology yielded full‐thickness skin as evidenced by clinical observation and skin biopsy 5 months after surgery, providing relief for debilitating split‐thickness skin graft contracture in a pediatric burn case.

## Graphical abstract

New autologous skin regeneration technology yielded full‐thickness skin as evidenced by clinical observation and skin biopsy 5 months after surgery, providing relief for debilitating split‐thickness skin graft contracture in pediatric burn case.

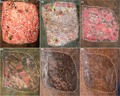

## 1 INTRODUCTION

Large deep partial‐thickness and full‐thickness cutaneous defects often require excision and split‐thickness skin grafting (STSG) because the surrounding intact skin and endogenous regenerative populations are unable to heal the defect. Donor site availability and contraction of the grafted wound are significant limitations for patients requiring extensive skin grafting. Other skin therapies commonly augment one or two components of the wound healing process, yet no adjunctive therapies utilize all components of wound healing or can regenerate full‐thickness skin. A recently developed, commercially available autologous homologous skin construct (AHSC) technology utilizes the patient's endogenous cutaneous regenerative potential to expand and generate full‐thickness skin with all epidermal and dermal components. The ability of this technology to regenerate full‐thickness skin following excision of a scarred STSG was evaluated. A 10‐year‐old boy who suffered a large upper‐torso burn wound treated with STSGs 1.5 years earlier developed painful and functionally limiting scar contractions. The provider, patient, and patient's guardian elected to excise the affected areas and undergo treatment with an AHSC. A 17.5 cm^2^ piece of full‐thickness skin was harvested from the groin and sent to a biomedical manufacturing facility and processed into AHSC the day following harvest. The AHSC was returned to the provider the following day and applied to a 200 cm^2^ wound immediately following excision. Tissue healing was monitored during routine follow‐up visits with digital photography and dermoscopy for 7 months. Histologic analysis was performed on a biopsy of the regenerated skin at 5 months. The AHSC demonstrated 100% graft take and initial epithelialization with repigmentation by 1 week postoperatively, progressing to complete epithelial coverage at 8 weeks with minimal contracture. At 5 months postoperatively, imaging and biopsy of the reconstructed site revealed regeneration of full‐thickness skin, including rete pegs, subdermal fat, and hair. At 11 months, the regenerated skin remained supple with restored ROM and without evidence of adverse scarring. The AHSC was able to expand and generate full‐thickness skin in a critical‐sized cutaneous defect in a case of pediatric burn reconstruction for debilitating STSG contracture.

The regenerative capacity of skin is well documented.^1^, ^2^ Complex niches of cells incorporating pluripotent stem cells, transient amplifying cells, and supporting stromal populations have been identified throughout the dermis including the hair follicle bulge, interfollicular epidermis, and sweat glands.^3^ These heterogeneous populations rely heavily on coordinated intercellular communication and dynamic interactions with their microenvironment and systemic environment for their maintenance and activation for wound repair.^4^ Once activated by injury, wound healing proceeds in a complex progression of cellular events commonly divided into four overlapping phases consisting of hemostasis, inflammation, proliferation, and maturation.^5^ An important subset of events occur throughout this process including fibroplasia, angiogenesis, and epithelialization.^6^ Critical‐sized defects occur when the defect is too large for the remaining endogenous cutaneous regenerative populations to repair the injury.

Autologous skin grafts have remained the standard of care for both cutaneous reconstruction and definitive wound coverage.^7^ These include the use of split‐thickness skin grafts (STSGs) and full‐thickness skin grafts (FTSGs). Although these grafts have provided good results in the majority of patients, both have intrinsic and extrinsic limitations (Table 1).^7^, ^8^, ^9^ STSGs will typically obtain the top 0.15‐0.3 mm of skin (epidermis) and exclude dermal appendages.^7^ As a result, STSGs lack the durability of native skin and do not include hair follicles, the regenerative stem cell niche of the follicular bulge, glandular structures, or hypodermal adipose populations.^10^, ^11^, ^12^ Due to the inability to regenerate full‐thickness cutaneous tissue, graft ischemia, and lower relative levels of elastin, STSGs often contract significantly following placement through a process called secondary contraction.^8^, ^11^ While large recipient surface areas can be covered with STSG techniques, donor sites are often large and painful and can be associated with a variety of morbidities such as scarring, abnormal pigmentation, chronic pain, and infection.^13^, ^14^

**Table 1:** Split‐thickness skin grafts (STSG) and full‐thickness skin grafts (FTSG)

Type of Skin Graft	Clinical Use	Positives	Negatives
Thin STSG	Burn wounds, chronic wounds, less‐vascularized wound beds, acute well‐vascularized wounds	Good graft take Fast donor site healing Ability to reuse prior donor site	Graft contraction and scarring Epidermis only, lack of functional dermal components
Thick STSG	Same as above	Good graft take Less secondary contraction as thin STSG Increased graft stability	Slower donor site re‐epithelialization
FTSG	Reconstruction of areas with of high cosmetic and/or functional demand (face, hand); well‐vascularized, noninfected wounds	Minimal secondary contraction Increased graft stability Contains all dermal components including hair and stem cell niches	Limited donor site availability and limited expansion capability Increased graft loss

Full‐thickness skin grafts (FTSGs) are intrinsically preferable to STSGs for autologous reconstruction because they contain all skin layers as well as the functional and regenerative appendages located within each cutaneous compartment. Thicker FTSGs are more resistant to shear forces, as they more readily maintain the native dermal‐epidermal interface with less contraction after application.^10^, ^11^ The primary limitations of FTSGs stem from lack of donor skin because the defect requires primary closure. In addition, FTSGs have higher metabolic demands carry an inverse relationship between skin thickness and oxygen perfusion capability. Therefore, FTSGs require wound beds with robust vascular supply and their application is typically limited to smaller wounds that benefit from full‐thickness skin functions such as the face, hands, and joints.

Given these limitations with traditional STSG and FTSG, a spectrum of commercial skin substitutes have been developed from a variety of synthetic, allogeneic, and xenogeneic sources.^15^ Although some of these substitutes are able to augment one or two components of the healing cascade including angiogenesis and epithelialization, none are able to recapitulate the complex cellular networks and interactions required to regenerate full‐thickness skin.^7^, ^9^ Recent advances in the understanding of molecular, cellular, and systems biology involved in wound repair suggest that utilization of limited cell types or components of skin regenerative systems are not sufficient for complex tissue repair and regeneration. A novel autologous cell‐tissue–based technology, recently made commercially available, leverages the endogenous regenerative potential of skin to regenerate full‐thickness, functional skin with dermal appendages including hair follicles and sweat glands and their associated stem cell populations following a single application.^16^

This autologous homologous skin construct (AHSC) is created from a small harvest of healthy skin full‐thickness skin from the patient. The harvest is sent overnight via commercial carrier to an FDA‐regulated biomedical manufacturing facility. The processing of AHSC involves the generation of microaggregates, which include the endogenous regenerative and supportive cells and tissues found within skin that are involved in native wound healing. In addition to activation of these regenerative populations, processing optimizes the surface area to volume ratio of AHSC, which improves its ability to be sustained by plasmatic imbibition within the wound bed. The product is not cultured ex‐vivo, rather it is returned within 14 days to the provider per their discretion and it is spread onto a freshly prepared wound bed and dressed, in a similar manner to a skin graft. The AHSC uses the innate supporting substrates generated by the wound bed to implant and expands, therefore closing the wound from the inside out.^16^

This report is an early clinical experience evaluating the ability of this novel technology to regenerative full‐thickness skin confirmed by skin biopsy in a critical‐sized full‐thickness cutaneous defect created after excising a functionally limiting scar resulting from STSG treatment of a burn injury in a 10‐year‐old boy.

## 2 METHODS

### 2.1 Patient presentation and operative assessment

**Figure 1:**
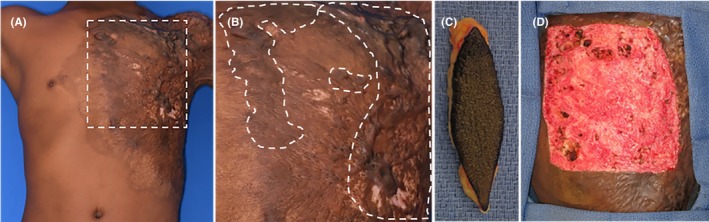
A, Preoperative images of anterior chest burn scar contracture, keloid scar, and previously placed split‐thickness skin graft with region of interest shown in (B). C, Harvested skin sent for AHSC processing. D, Full‐thickness wound bed preparation for application of AHSC

A 10‐year‐old African‐American boy developed progressive chest scarring, contraction, and keloid formation associated with chronic pain and range of motion (ROM) limitations following treatment with a STSG 1.5 years prior for a full‐thickness open flame burn injury involving his left chest, back, axillary, upper extremity, and neck (Figure 1A and 1). Staged operative reconstruction of his left elbow and axillary contractures with adjacent tissue transfers were planned. Options for treating the scar tissue of the left chest were presented to the patient and his family, which included serial steroid injections, serial laser treatment, excision, and repeat STSG, excision with Integra^®^ Bilayer Wound Matrix (Integra LifeSciences) placement followed by STSG, tissue expander placement with FTSG, and/or application of the autologous tissue product (SkinTE™; PolarityTE, Inc). After an extensive discussion regarding the novelty of the treatment, the patient's parents signed a written informed consent to proceed with the AHSC treatment despite the lack of long‐term human outcome data. Written informed consent was also granted by the patient's parents to follow healing progression and therapeutic outcomes with digital photography and video. The Louisiana State University Health Science Center IRB (#10326) approval was obtained for this study. This study meets the waiver criteria described in 45 CFR 164.512 (i) (2) (ii). All methods were carried out in accordance with relevant guidelines and regulations.

### 2.2 Operative procedure

At the time of elbow contracture release and adjacent tissue transfer, a 17.5 cm^2^ elliptical full‐thickness skin harvest was taken from the left abdomen immediately inferior to the area of scarring and sent to the manufacturer (Figure 1C). The donor site was closed primarily. Per clinical request for procedure scheduling, the AHSC was returned 48 hours after harvest and deployed into a 200 cm^2^ full‐thickness defect over the left chest, where a region of the contracted and scarred STSG had been directly excised from the chest wall (Figure 1D). The defect to harvest ratio in this case was 11.4:1. The product was applied evenly throughout the excised area using the flat backside of a forceps and spreading the product in a manner similar to that for STSG application. The wound was dressed with sterile silicone, petroleum gauze, bacitracin, and an elastic bandage overlay. The patient was discharged home the day of surgery, and dressings were changed at weekly intervals. Graft take and healing were photographically documented using digital single reflex photography (Canon) and dermoscopy (Vectra; Canfield Scientific).

### 2.3 Tissue biopsy and polarized dermoscopic imaging

Five months following graft application of the AHSC, a biopsy of the AHSC‐regenerated tissue was performed. During the biopsy procedure, macroscopic digital and polarized dermatoscopic imaging was obtained in order to assess melanin organization within the skin and identify hair follicles within the tissue. At the time of biopsy, the healed tissue graft was “pinch tested” while being video recorded.

### 2.4 Stereomicroscopy

The skin biopsy specimen from the patient was whole‐mount imaged using a Leica M205 FA stereomicroscope (Leica Microsystems) to identify gross tissue features and measurements.

### 2.5 Ancillary staining

Skin specimens were preserved in 10% normal buffered formalin (NBF), paraffin embedded and sectioned into 6 micrometer thick specimens for ancillary histological staining. Hematoxylin and eosin, trichrome, and periodic acid‐Schiff stains were used to examine the relative microanatomy of the biopsied tissue.

### 2.6 Outcome and follow‐up

No postoperative complications, infection, or graft shearing was identified at the recipient site following application of the autologous tissue product. No donor site complications or dehiscence were observed and the primarily closed donor site healed in a linear orientation without adverse scarring.

**Figure 2:**
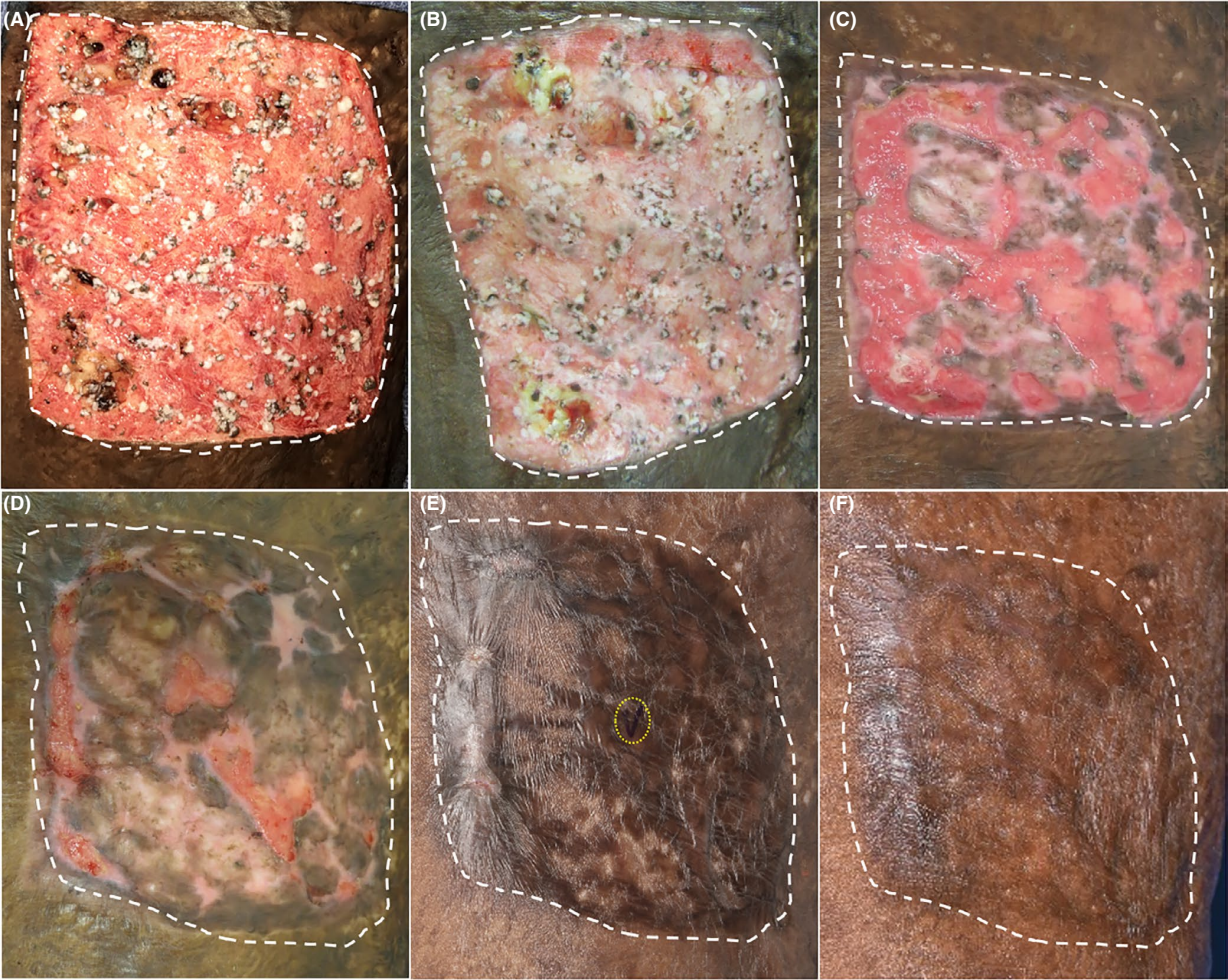
Linear progression of wound healing following product application. A, Day of deployment—AHSC is spread evenly over the debrided 200 cm^2^ wound, B, 2 wk post‐AHSC deployment, C, 4 wk, D, 6 wk, E, 5 mo, black pen mark (circled in yellow) indicates site of biopsy, and F, 13 mo. Area treated with AHSC highlighted with white dotted lines

Sequential imaging of the region depicts progressive propagation and expansion of the autologous tissue from discrete enlarging foci throughout the wound bed over time (Figure 2). Graft take, epithelialization, and partial repigmentation were evident 1 week postoperatively and progressed to complete full‐thickness skin coverage at 8 weeks with minimal contracture and scarring at the interface between the regenerated skin and surrounding native skin at 5 months (Figure 2E). At a durability follow‐up visit, 13 months post‐AHSC therapy, contraction had not recurred, and the wound site was completely healed with regenerated skin continuing to normalize to the surrounding healthy tissue (Figure 2F). Dressings were discontinued at 8 weeks once complete epithelialization was visibly apparent. A single application of AHSC was used with no additional interventions or products beyond standard dressing changes were needed during the postoperative period.

**Figure 3:**
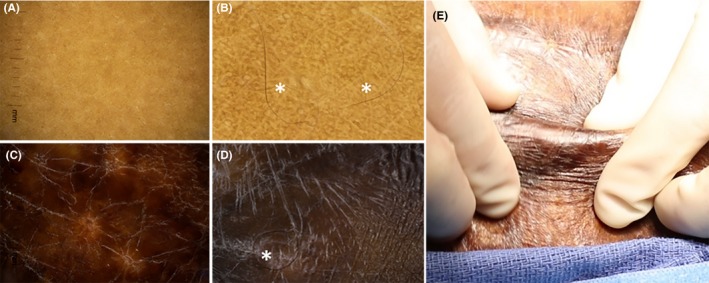
Polarized dermoscopic imaging of native skin (A,B) and AHSC‐regenerated skin (C,D). High‐powered fields of native skin (B) and AHSC‐regenerated skin (D) demonstrated distribution of melanin and retaining darker pigment of harvested skin. Hair (*) in AHSC‐regenerated skin (D) resemble those of native skin (B). E, Pinch testing of AHSC‐regenerated skin demonstrated pliable, noncontracted skin with functional characteristics similar to native skin

Polarized dermoscopic imaging was performed at 5 months on both native and AHSC areas and compared visually (Figure 3). Distribution of melanin and melanocyte populations were observed to be confluent under polarized dermoscopic evaluation of the regenerated skin but appeared to exhibit less random orientation when compared to native skin. Hair follicles were also identified in the regenerated skin that appeared similar to native hair follicles in both structure and pigment (Figure 3B and 3D). The patient reported sensation to touch of the AHSC‐regenerated area and expressed that the treated area had an improved appearance and movement. Pinch testing revealed that the healed skin was pliable, and supple suggesting that there was an adequate hypodermal adipose layer providing both cushioning and gliding (Figure 3E, Video S1).

**Figure 4:**
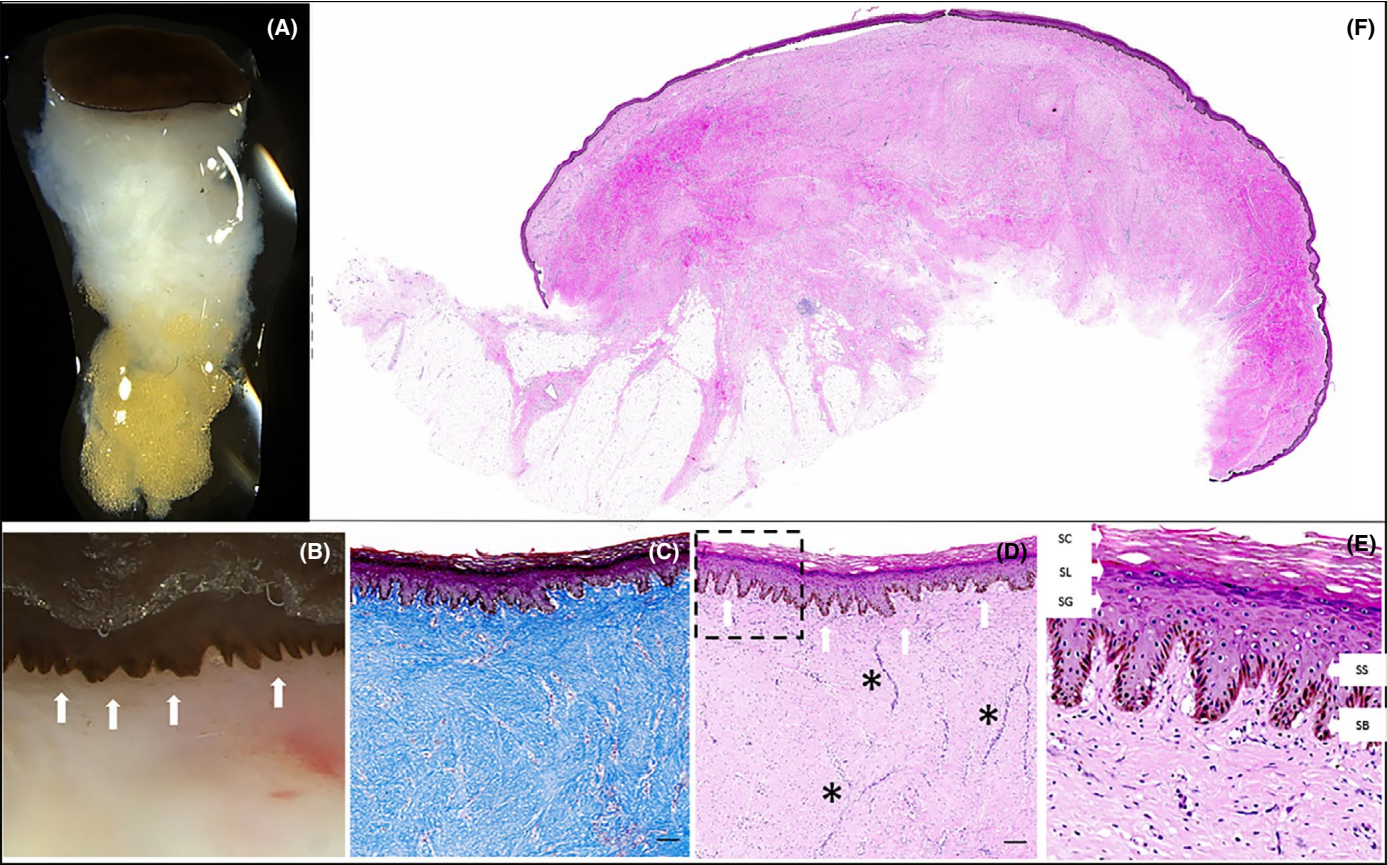
A, Stereoscopic image of punch biopsy taken at 5 mo demonstrating recapitulation of full‐thickness skin including epidermis, dermis, and hypodermal fat. B, High‐power field shows rete pegs at epidermal‐dermal junction (white arrows). C, Masson's trichrome staining demonstrating normal collagen architecture. D, Hematoxylin and eosin staining demonstrating full‐thickness skin, rete pegs (white arrows), and dermal vasculature (*) with region of interest E, showing all layers of the epidermis (SC, stratum corneum; SL, stratum lucidum, SG, stratum granulosum; SS, stratum spinosum; and SB, stratum basale). Scale bars represent 100 micrometers. F, Hematoxylin and Eosin staining of the whole section of regenerated skin biopsy demonstrating full‐thickness skin with epidermal, dermal, and hypodermal layers present 5 mo post‐AHSC therapy

Stereoscopic imaging of the biopsy performed at 5 months post‐AHSC application demonstrated recapitulation of the epidermis, dermis, and hypodermal fat layer (Figure 4A and 4B). Ancillary staining of the biopsy demonstrated regeneration of full‐thickness skin with skin architecture that included a fully developed stratified epidermis, epidermal/dermal junction with rete pegs, papillary and reticular dermal compartments as well as an integrated organized dermal plexus vasculature (Figure 4C and 4D).

## 3 DISCUSSION

Skin's role as a protective barrier exposes it to physical trauma and injury requiring robust systems of tissue maintenance and regeneration.^3^ Comprised of cells originating from all three germ‐layers, skin contains several regenerative multicellular niches located throughout the dermis and epidermis, notably the interfollicular epidermis, hair follicle bulge, and sweat glands.^1^, ^3^ Signaling cascades involved in injury and hemostasis activate these stem cell populations, which can regenerate all cellular components of full‐thickness skin.^1^ Although existing advanced skin substitutes may contain one or two cellular populations of the skin, typically keratinocytes and/or epithelial cells combined with static engineered scaffolds, none contain all of the components or the necessary cellular populations required to regenerate full‐thickness skin and its associated appendages.

Taking advantage of the endogenous regenerative elements of skin, a novel autologous homologous skin construct has been developed and manufactured using a full‐thickness skin harvest from an area of uninjured skin. The manufacturing event involves segmentation of the input material and activation of the innate stem cells and their supportive niche in a similar fashion to the activation that occurs following skin injury.

By deploying the AHSC into a wound bed, the regenerative cellular populations supported by their conserved supporting microenvironment are optimized to survive on plasmatic imbibition required by all transferred tissues during early graft take due to their increased surface area to volume ratio. Supported by the body's natural repair media created by the wound bed itself and not exogenous media and in vitro culturing conditions, the AHSC expands within the wound, effectively healing it from the inside out.

Although currently recognized definitions of wound healing focus on complete epithelialization, it is well understood that more than superficial epithelialization is required for complete wound healing. By regenerating the dermal appendages including hair follicles and sweat glands as well as the hypodermal fat, AHSC treatment results not only in complete wound coverage but recapitulation of the many different functions skin provides.

This novel technology was evaluated in a pediatric case of burn reconstruction that highlights issues with STSG, namely contraction and adverse scarring. A single application of AHSC demonstrated stable and progressive wound coverage, minimal contracture, and full‐thickness skin architecture on dermoscopic imaging and tissue biopsy at 5 months. Use of this tissue product, including the skin harvest, manufacturing, and application, fit within the standard wound management workflow and required no additional product‐specific medical device for application or postoperative management. As a child, this patient required general anesthesia for skin harvest; however, this could be performed under local anesthesia and in a clinic setting similar to a skin biopsy or excision.

Additional studies, including randomized controlled trials, are required to further evaluate the applicability and limitations of this novel technology. Conventional wound healing progression indicates that wound remodeling occurs over the course of an entire year and therefore we expect that the region will continue to change over time in both pigment and directionality of the underlying extracellular matrix. Although this early clinical use is encouraging, it is unclear how AHSC may perform in other wound classes including acute burns, chronic wounds, and complex wounds with exposed deep structures, and in less healthy patients with comorbidities and systemic conditions.

## 4 CONCLUSIONS

AHSC’s cooptation of the body's regenerative capacity by preserving key cellular populations and their interdependent relationships is a novel approach to treating cutaneous defects. By avoiding excessive tissue processing and exogenous engineering that can result in cellular senescence, it resulted in successful regeneration of full‐thickness autologous hair‐bearing skin in a manner consistent with native tissue repair in a complicated pediatric wound.

## CONFLICT OF INTEREST

Dr Mundinger received consulting fees from PolarityTE and is on the company's clinical advisory board. No other author has any conflict of interest to disclose, and there has been no financial support for this work, use of the technology, or manuscript preparation. Commercial Disclosure: G. Mundinger is a Clinical Advisor for PolarityTE.

## AUTHOR CONTRIBUTIONS

CP and GM: served as the principle surgeons and wound care providers for this case, wrote the original draft, constructed the figures, and provided multiple revisions of all materials. MS: provided histological processing and analysis of resultant specimen as well as contributed to multiple revisions of the manuscript as well as consulted on the pathological findings of this case. SS: facilitated the logistics and conduction of this study as well as contributed to the multiple revisions of this manuscript.
